# Hantavirus infection in central Sri Lanka – an unusual clinical presentation: a case report

**DOI:** 10.1099/acmi.0.000554.v3

**Published:** 2024-04-15

**Authors:** T. T. Pattiyakumbura, S. H. Pathirathne, M. A. R. V. Muthugala

**Affiliations:** 1 National Hospital Kandy, Kandy, Sri Lanka; 2 Provincial General Hospital Badulla, Badulla, Sri Lanka

**Keywords:** Asia, Atypical presentation, hantavirus cardio-pulmonary syndrome, hantavirus infection, Puumala/ Puumala-like virus, Sri Lanka, Zoonoses

## Abstract

Hantavirus infections are emerging zoonoses. In Asia, the hantavirus commonly manifests as haemorrhagic fever with renal syndrome (HFRS), apparent with fever, thrombocytopenia and acute kidney injury. There are a few cases with the atypical clinical course with cardiopulmonary symptoms in Asia including Sri Lanka. Here, we report a case of hantavirus infection with an atypical cardiopulmonary syndrome-like illness with serological evidence of the Puumala/Puumala-like virus.

## Data Summary

No data was generated in this case report.

## Introduction

Hantavirus infections are emerging zoonoses, hantaviruses belonging to the genus *Orthohuntavirus*, the family *Hantaviridae*, order Bynyavirales [1]. Hantaviruses cause two different types of acute febrile illness, namely haemorrhagic fever with renal syndrome (HFRS) in Euro-Asia, and Africa, and hantavirus cardio-pulmonary syndrome (HCPS) in America [2]. Infections with atypical or mixed clinical features are also reported worldwide [3]. Sri Lanka is an endemic country for both leptospirosis and hantavirus, the majority of clinical cases of hantavirus infection are probably misdiagnosed as leptospirosis because they both share similar clinical and epidemiological features [4]. In Asia, hantavirus commonly manifests as HFRS, which includes headaches, backaches, abdominal pain, fever, chills, nausea and impaired vision; subsequent symptoms include low blood pressure, acute shock, vascular leakage, thrombocytopenia and acute kidney failure [2]. We present an atypical case of a young female from the central part of Sri Lanka, who presented with pulmonary symptoms and was eventually diagnosed with hantaviral infection with an atypical cardiopulmonary syndrome-like illness.

## Case presentation

A 21-year-old lady was admitted to a tertiary care hospital in the central part of Sri Lanka, with symptoms of sudden onset shortness of breath on exertion and fever for 5 days duration. Furthermore, her symptoms were associated with arthralgia and myalgia. She has been experiencing worsening symptoms at night with sleep disturbance. She was a university student and had visited a paddy field, 2 weeks prior to the onset of symptoms.

On examination, she was mildly dyspneic and febrile. She was not icteric or pale. The respiratory system examination revealed an increased respiratory rate of 28 cycles per minute. The oxygen saturation was 93 %, and the lung auscultation revealed bibasal crepitations, with reduced breath sounds in the right middle zone. At the time of admission, cardiovascular, abdominal and neurological examinations were unremarkable.

**Fig. 1.**
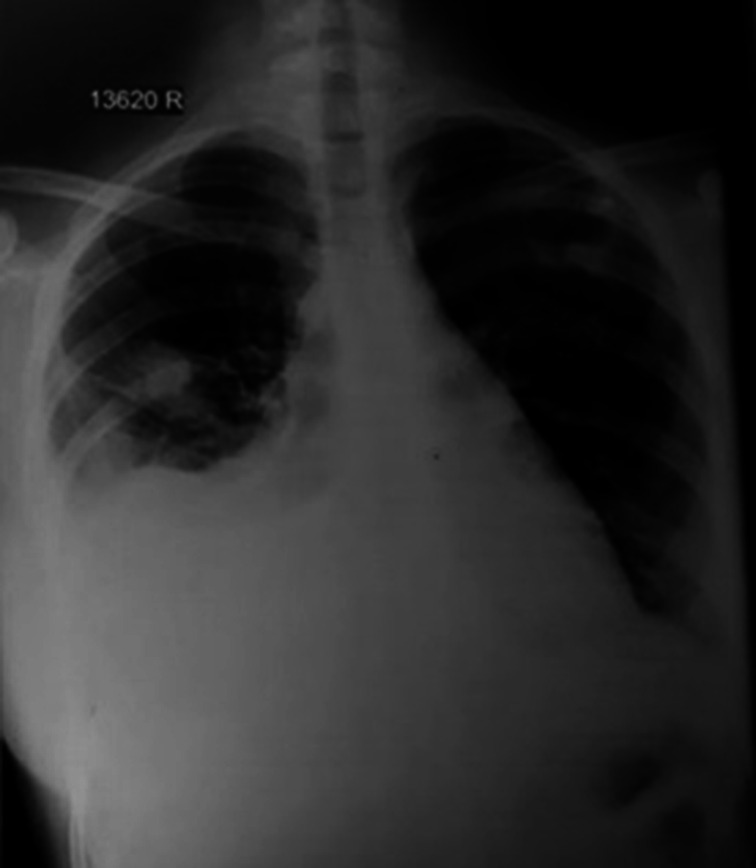
Chest radiograph-bilateral pleural effusion with opacification in the right mid-zone.

**Fig. 2.**
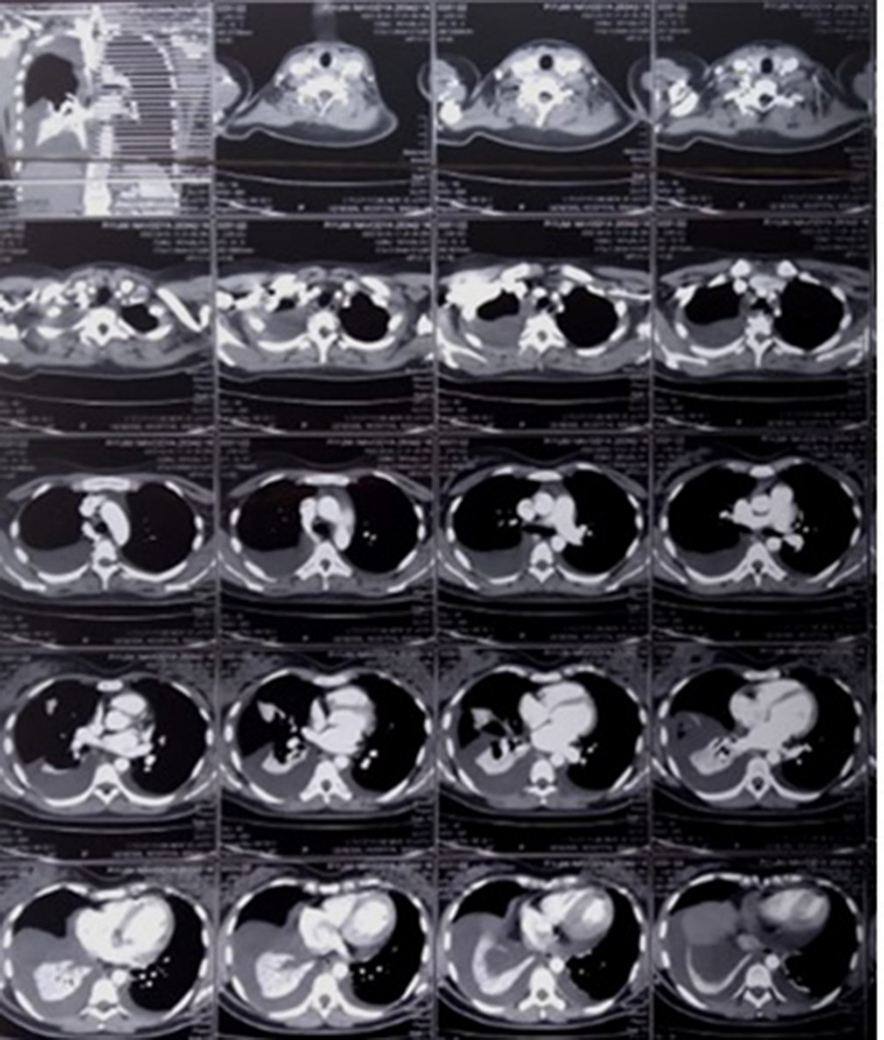
CECT chest-collapsed consolidation of the right middle lobe of the lung with bilateral pleural effusions.

Laboratory investigations showed marginal leukocytosis, 11.4×10^9^ l^−1^ with marginal lymphocytopenia. Haemoglobin level was 11.3 g dl^−1^, platelet count was 2.8×10^9^ l^−1^, and C-reactive protein level was not elevated. The liver and renal function were within the normal range. The chest radiograph showed mild to moderate bilateral pleural effusion with opacification in the right mid-zone (Fig. 1). The contrast-enhanced chest-computed tomography (CECT) further confirmed collapsed consolidation of the right middle lobe of the lung with bilateral pleural effusions (Fig. 2). The electrocardiogram showed diffuse T inversion in Antero-lateral leads, however, the troponin I was negative. The initial two-dimensional echocardiography showed a mild to moderate pericardial effusion and the ejection fraction was more than 60 %.

**Fig. 3.**
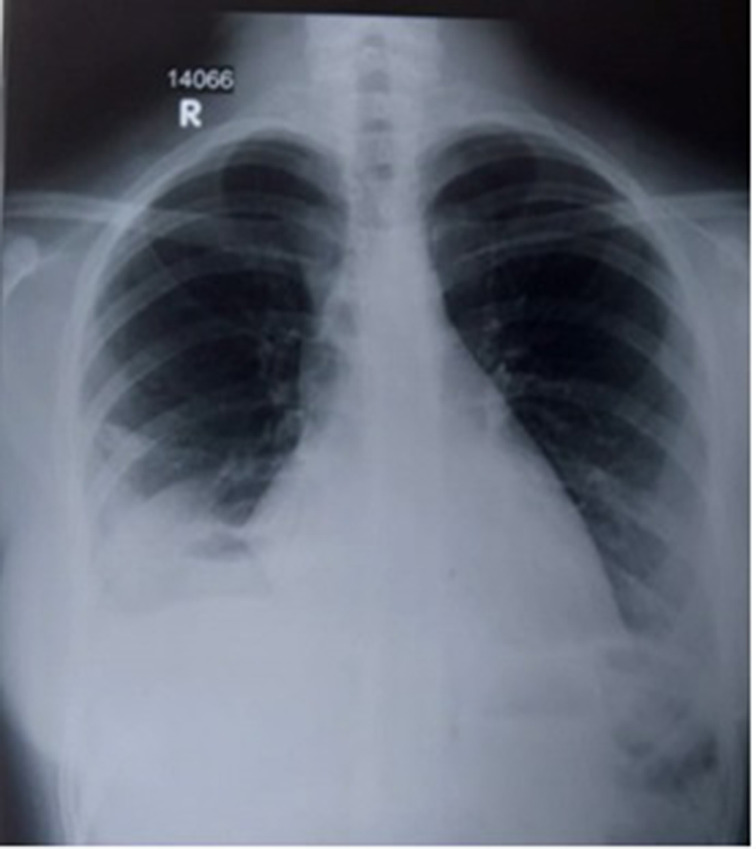
Post-aspiration chest radiograph.

Therapeutic aspiration of 500 ml of right pleural effusion was performed after day 2 of the admission (Fig. 3). Pleural fluid was investigated for malignant cells, gram stain and culture, and mycobacterium tuberculosis. No significance was detected in those investigations. SARS-CoV-2 infection was excluded as a local policy on admission with a real-time PCR test. A bronchoscopy was performed in view of the collapsed right middle lobe. It did not reveal any endobronchial lesion. Bronchoalveolar lavage was sent for microbiological and pathological evaluation, which eventually became negative. Leptospirosis was excluded with IgM enzyme-linked immunosorbent assay (ELISA) (Standard Diagnostics, Yongin Si, South Korea) on acute and convalescent serum samples. Dengue-specific IgM/IgG was not detected on day 7 of the illness.

**Fig. 4.**
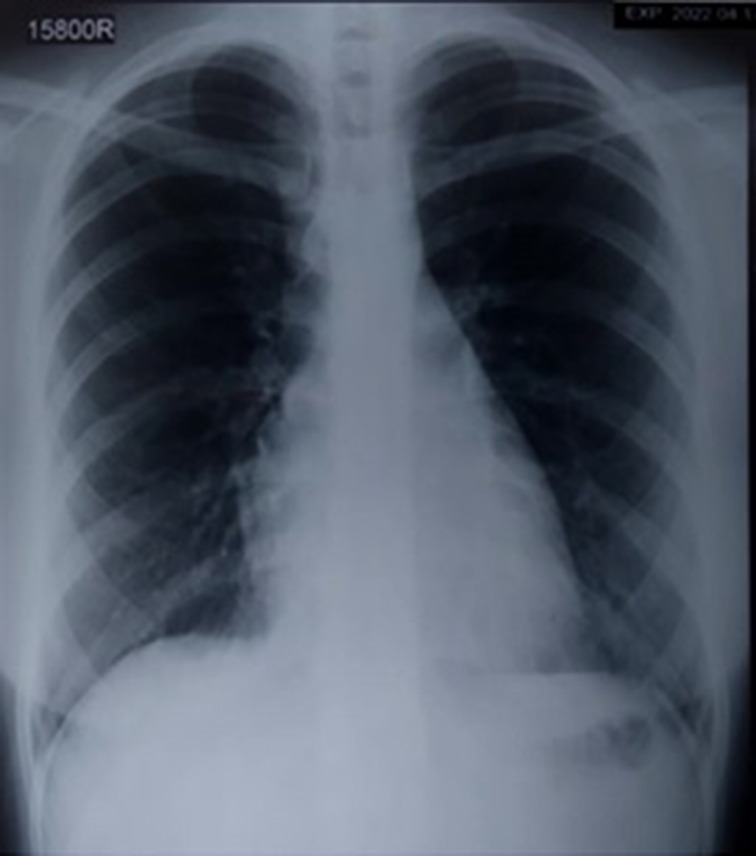
Chest radiograph after 1 month of recovery.

Meanwhile, the patient was treated for atypical pneumonia with oral azithromycin and oral co-amoxiclav for 14 days. Additionally, the patient was given supportive care with oxygenation and hydration and discharged from the hospital, on day 5 of admission. On discharge, a serum sample that was collected (on day 10 of the illness) was sent for the detection of hantavirus antibodies. Hantavirus-specific IgM antibodies were detected by using a locally validated commercial ELISA (Anti-Hanta Virus Pool 1’ Eurasia’ ELISA (IgM), Germany). The patient was contacted for subsequent testing and a convalescent serum sample was obtained; an eightfold rise in hantavirus-specific IgG levels in acute and convalescent samples by commercially validated in-house immunofluorescent assay (IFA) confirmed the hantavirus infection (PROGEN, PROGEN, Biotechnik GmbH, Germany). Additionally, the acute serum sample was subjected to the immunoblotting test (Mikrogen diagnostik, Germany). It revealed the patient had been infected with the Puumala or Puumala-like virus. The patient had an uneventful recovery (Fig. 4).

## Discussion

This patient was a previously healthy individual. She presented with cardio-pulmonary symptoms, as well as arthralgia and myalgia, with the background of rodent exposure 2 weeks prior to developing symptoms. However, she had a near-normal full blood count and inflammatory markers suggesting that this was an atypical lung infection. Although extrapulmonary signs of atypical pneumonia such as hepatitis, myocarditis and renal injury are well-known, her renal and liver function tests were against extrapulmonary manifestations [5]. Sri Lanka is a hyperendemic country for dengue infection, dengue was also excluded [6]. The patient was started on oral azithromycin and oral co-amoxiclav to treat against pathogens that can cause both atypical pneumonia (Mycoplasma, Chlamydia, Legionella, Rickettsia) and common community-acquired infections [7].

Meanwhile, 500 CC of pleural fluid was drained from the right side of the lung during a therapeutic pleural tap. With molecular methods (gene-Xpert) and culture methods, pulmonary tuberculosis was ruled out, which has a moderate prevalence in Sri Lanka [8]. Considering the clinical and radiological findings, rare possibilities of lung malignancy and lymphoma were also ruled out. Since the pleural fluid and blood for other bacterial cultures were negative, the clinical team focused on infections that are rarely reported in Sri Lanka but can resemble a similar clinical picture. This decision was supported by the appearance of mild to moderate pericardial effusion with features of myocarditis, in the two-dimensional echocardiography. As a result, a serum sample was collected for hantavirus serology, which was shown to be positive for hantavirus-specific IgM, and a fourfold rise in hantavirus-specific IgG was demonstrated confirming the diagnosis [9]. The retrospective diagnosis was made as a hantavirus infection with HCPS-like presentation leads to the capillary leak into the pulmonary bed causing pulmonary oedema, pleural and pericardial effusion, and myocarditis [10].

In Asia, patients with fever of unknown aetiology had been reported as having serologic evidence of hantavirus infection [11]. Also, there are cases of previously healthy travellers has developed Hantavirus pulmonary syndrome immediately after arriving in Europe from Southeast Asian countries [12]. However, HCPS-like presentation is not common in the Asian region, because the Puumala virus and its host are confined to Europe. However, many Asian countries including Sri Lanka and India had reported fatal leptospirosis-like cases caused by unexplained PUUV-like viruses [13]. Therefore, this could be due to a novel Puumala-like virus or a virus that serologically cross-reacts with the Puumala virus. Dalugama *et al*. discussed a similar case in Sri Lanka presenting clinical features suggestive of leptospirosis, eventually diagnosed as hantavirus infection. However, in that case, the patient had a mixed clinical picture suggestive of renal and cardiopulmonary symptoms and had been treated with broad-spectrum antibiotics and methylprednisolone for suspected leptospirosis infection [14]. Ehelepola *et al*. also described two cases, where both patients had a combination of pulmonary and renal involvement similar to the patient described by Dalugama *et al*. and diagnosed as having atypical hantavirus infection [15]. In our case, this patient was reported from a geographic area where no patients had previously been identified for hantavirus infection [4]. The patient had predominant cardiopulmonary symptoms that were extensively investigated for atypical pneumonia without prominent renal involvement. Also, the patient was not treated with methylprednisolone as her initial clinical diagnosis was atypical pneumonia. We further demonstrated an eightfold rise in hantavirus-specific IgG levels in this patient using her acute and convalescent samples by commercially validated in-house IFA. Also, we were able to identify the responsible hantavirus type using the acute serum sample in which the patient was found to be infected with the Puumala or Puumala-like virus. However, hantavirus confirmatory tests and typing were not attempted in other cases described by Dalugama *et al.* and Ehelepola *et al.*

Hantavirus has a wide range of hosts including rodents, shrews and moles [16]. Inhalation of aerosols contaminated with infected rodents' faeces, urine or saliva, or, rarely, direct contact with infected rodents' faces or urine, or, a bite from an infected rodent means that humans always acquire the virus [17]. Hantavirus was initially identified in Sri Lanka in 1986 when four patients out of 248 screened with leptospirosis-like illness tested positive for the hantavirus [16]. Since then, patients have tested positive for the Seoul (SEOV), Hantaan (HTNV), Puumala(PUUV), Puumala-like, and Thailand virus-like viruses (THAIV) [4,16]. During the years 2013–2015 and 2016–2018, patients with hantavirus cardiopulmonary syndrome-like infections were documented in the north-central part of Sri Lanka [4]. Even though the number of reported cases in HCPS is much lower than in HFRS globally, the average case fatality is around 40 %, which is significantly higher than in HFRS [9]. The clinical course of HCPS is divided into three stages: prodromal, cardiopulmonary and convalescent, with symptoms ranging from mild hypoxia to respiratory failure with cardiogenic shock. Fever, chills, muscle aches, headaches, dizziness, nausea, abdominal pain, vomiting, anorexia and diarrhoea characterize the prodrome phase of infection, which is followed by the cardiopulmonary phase, which results in capillary leakage and pulmonary oedema. Then the convalescent period follows, during which the patient continues to recover from the disease [1]. Clinical and epidemiological data, as well as laboratory tests, are used to diagnose HFRS and HCPS. However, the early indicators of the disease are non-specific, diagnosing hantavirus infections solely on clinical grounds is difficult, especially in cases with mild to moderate clinical symptoms. Serological tests such as IgM-ELISA, and IgG fourfold rise in acute and convalescent samples are the most extensively used approach for HFRS and HCPS diagnosis, and they have good sensitivity and specificity [1]. Indirect immunofluorescence assays are also commonly used in diagnostics [18]. In addition, commercially validated ELISA and immunochromatographic IgM-antibody tests have also been developed [19]. Importantly, the virus is rarely detectable in blood at the onset of symptoms, limiting the diagnostic use of molecular assays at this stage [1].

There is currently no Food and Drug Adminsitration-approved medicine for HFRS and HCPS; treatment is mostly supportive. Haemodynamic and pulmonary support is provided; supplemental oxygen should be supplied to hypoxic patients and patients with severe diseases might need mechanical ventilation. The use of intravenous ribavirin to treat has been studied. However, in limited studies, ribavirin showed no therapeutic effect on the patients [20]. Many seroprevalence studies show that hantavirus infections among farmers and/or forestry workers are occupationally related because those places create a high chance of human–rodent contact [21]. According to a mammalian study conducted in Sri Lanka, certain types of rodents contribute to the maintenance and transmission of leptospirosis and the hantavirus in Sri Lanka. In that study, the majority of the rodents had antibodies that reacted with orthohantavirus antigens related to PUUV and/or SEOV [22]. Hence, exposure to rodents and their excrement should be avoided to prevent the transmission of hantavirus infection [1].

In Sri Lankan patients with clinical symptoms suggestive of leptospirosis and patients with a fever of unknown aetiology, hantavirus infection should be considered as a differential diagnosis. In addition, diagnostic facilities in hospitals should be expanded to assist clinicians in early disease diagnosis. Public awareness about the disease and how to avoid rodent exposure should be raised as a preventative step. Although HPCS has been typically discovered in the Americas, the HCPS-like infection reported in Sri Lanka could be due to a novel virus that requires further investigation to uncover the exact virus.

## Abbreviations

CECT: contrast-enhanced chest computed tomography
ELISA: enzyme-linked immunosorbent assay
HCPS: hantavirus cardio-pulmonary syndrom
HFRS: haemorrhagic fever with renal syndrome
IFA: immunofluorescent assay
